# Implementation of personalized self-management support using the self-management screening questionnaire SeMaS; a study protocol for a cluster randomized trial

**DOI:** 10.1186/1745-6215-14-336

**Published:** 2013-10-17

**Authors:** Nathalie Eikelenboom, Jan van Lieshout, Michel Wensing, Ivo Smeele, Annelies E Jacobs

**Affiliations:** 1 Radboud University Medical Centre, IQ healthcare, P.O. Box 9101, 114 6500 Nijmegen, HB, The Netherlands; 2 DOH care group, P.O. Box 704, Eindhoven 5600, AS, The Netherlands

**Keywords:** Self-management, Self-management support, Personalization, Chronic diseases, Primary care, Chronic care, Personalized medicine

## Abstract

**Background:**

The number of patients with one or more chronic diseases is rising. In several standards of care there is a focus on enhancing self-management. We applied the concept of personalization on self-management support and developed a self-management screening questionnaire (SeMaS). The main research objective is to assess the effectiveness of the SeMaS questionnaire and subsequent personalized self-management on patients’ self-management behaviors.

**Methods/Design:**

A cluster randomized controlled trial will be set up in 15 general practices in the Netherlands. The practices are all group practices, and member of one care group. The practices will be assigned to the control or intervention arms by stratified randomization. The strata are determined by the participation of the practice nurses in a course for behavioral change, and the nurse’s workload. Patients can be included if they are over 18 years of age, have at least one chronic condition and have a checkup appointment with the practice nurse in the inclusion period. The intervention consists of screening patients with the SeMaS questionnaire, producing a graphic profile with the abilities or barriers for self-management. Patients will receive tailored feedback. Practice nurses are trained in using the profile to enhance self-management of the patient and provide personalized self-management support. The use of individual care plans and self-management interventions is stimulated. In the control arm patients will receive care as usual. Patients of both trial arms will be asked to fill in the SeMaS questionnaire and additional questionnaires at inclusion and after 6 months. The primary outcome is the difference in the level of patient activation (PAM-13) between baseline and 6 months. Secondary outcomes include patient measures for lifestyle factors (exercise, diet, smoking), and process measures from medical record data analysis.

**Discussion:**

This manuscript presents the protocol for a cluster randomized clinical trial of personalized self-management support using the SeMaS questionnaire in chronically ill patients in primary care. By carrying out this study, scientific evidence is built for the effectiveness of personalized self-management support.

**Trial registration:**

The Netherlands National Trial Register: NTR3960.

## Background

The number of patients with one or more chronic conditions is vastly rising worldwide. In the United States, 133 million people suffered from one or more chronic disease in 2005 [1]. Hoeymans *et al*. stated that in the Dutch population 4.5 million people (28%) suffer from at least one chronic disease [2]. Tacken *et al*. showed that the percentage of patients who suffered from at least one chronic disease rose from 12.6% in 2003 to 15.0% in 2009 [3]. This can be explained by ageing of the population. The increase in number of patients with one or more chronic diseases will increase the workload in primary care, and increase healthcare costs. Chronically ill patients usually have several consultations with their general practitioner (GP) or practice nurse each year.

To increase quality of care, the quality of life of chronically ill patients, and to sustain healthcare costs, there is a large focus on enhancing self-management in people with chronic diseases in current guidelines and standards of care [4-6]. These guidelines are often based on the chronic care model of Wagner *et al*., a framework for integrated, patient-centered care [7]. The Department of Health of the United Kingdom defined self-management as ‘the care taken by individuals towards their own health and well being: it comprises the actions they take to lead a healthy lifestyle; to meet their social, emotional and psychological needs; to care for their long-term condition; and to prevent further illness or accidents’ [8]. Following this definition, self-management also means that the patient takes more responsibility for his or her own health.

Self-management programs have been developed for various chronic diseases and lifestyle changes [9-12]. Several studies have shown positive effects of these programs on clinical outcome measures, as well as patient-related outcome measures, such as participation, knowledge or activation [13-18]. These studies identified several factors that influence the probability of successfully completing the intervention, such as social support or self-efficacy.

**Figure 1:**
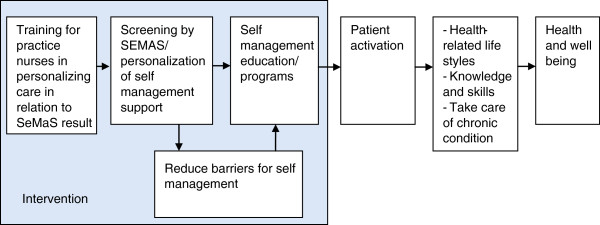
Logic model of the hypothesized effects of self-management screening questionnaire (SeMaS).

In other fields of research, especially human genetics, the concept of personalized medicine has been introduced [19]. In the field of genetics, patients are genetically screened, and subsequently receive treatment that is adapted to their genomic profile. In psychology, treatment is adjusted to psychological characteristics and thus, person-centered [20-22]. We applied the concept of personalization to self-management support. Here, personalization is defined as adjusting the self-management support to the individual characteristics of the patient. For example, in the case of low self-efficacy, the practice nurse and patient make an individual care plan with small steps in goal setting to enhance the self-efficacy of the patient. Current generic self-management measures include the patient activation measure (PAM-13), which measures the current level of self-management and the Health Education Impact Questionnaire (heiQ), measuring the outcome of an intervention or education program [23,24]. However, there was no generic instrument available to identify factors that could hinder successful self-management. For this purpose, we developed a self-management screening questionnaire (SeMaS), as described below. Providing patients with personalized self-management support, we expect the patient-activation level to increase, which subsequently has a positive effect on health-related lifestyles, knowledge, skills, and the ability to take care of the chronic condition. This in turn will positively influence overall health and wellbeing. The SeMaS questionnaire will provide specific information about the abilities and possible barriers for self-management. Using a manual, the healthcare provider will have a starting point to influence possible barriers and stimulate self-management. This is shown in the logic model in Figure 1.

The main research objective is to assess the effectiveness of the SeMaS questionnaire and subsequent personalized self-management support on patients’ self-management behaviors.

## Methods/Design

We will conduct a two-arm practice-level randomized trial with a postponed intervention in the control arm. The practice nurses will be trained in the SeMaS method. Since practice nurses were trained in the SeMaS method, the intervention was implemented at general practice level instead of patient level. Another problem in this study was the risk of contamination when control patients would be exposed to elements of the intervention. Therefore, randomizing all patients within a general practice (representing a cluster) to either the intervention or the control was the most logical choice.

### Practices

For this study, we recruited the practices through the primary care cooperation *De Ondernemende Huisarts* (DOH), or The Innovative General Practitioner. DOH is an innovative organization led by GPs who have a subspecialization. The care group comprises 15 general practices in the southeast part of the Netherlands, varying in size and degree of urbanization. Half of the practices are situated in the city, while the other half are located in surrounding villages. The context of this care group provided a well-developed infrastructure to perform this study. Together the practices serve approximately 115,000 patients who are registered at the practice according to the Dutch capitation system. The practices are group practices, in which groups of GPs work together, and are supported by practice nurses (64 GPs and 54 nurses in total). These nurses usually perform planned checkups following treatment protocols, while the GPs perform the annual checkups for chronically ill patients.

DOH has formulated a policy agenda for self-management in chronic patients [25]. The cooperation offers several self-management interventions or types of self-management support that are evidence-based as far as possible. The program of interventions consists of group courses, including the course ‘Beyond good intentions’, and group courses for smoking cessation, and an exercise project, KICK, which guides patients to local sport unions via physiotherapists [18,26].

The eHealth interventions consist of the Diabetes Interactive Education Program (DIEP), an Internet decision-aid for smoking cessation, patient education via informative websites about asthma and chronic obstructive pulmonary disease (COPD) from the Lung Foundation Netherlands and exercise (30minutenbewegen.nl), and a patient portal with options for self-monitoring and digital coaching on exercise, diet and smoking cessation [27].

The GPs and practice nurses have had training in motivational interviewing and the approach known as actual practice and maintenance, consisting of a behavior change model (referred to as a series of steps) and so-called person-related factors to enhance self-management [28,29]. Motivational interviewing is a client-centered counseling technique to facilitate and engage intrinsic motivation of the patients in order to change behavior. It has been found to be effective in several studies [30-32].

All 15 general practices of the DOH will participate in the trial. We recruited the practices through the DOH cooperation. The practice nurses will receive training in working with the SeMaS. As mentioned, we will perform randomization at the cluster level. Furthermore, as the ability of the practice nurse to influence the self-management behavior of the patient can affect the study results, we dichotomized practices into two equal groups using the percentage of practice nurses in the practice that participated in the behavioral change training [29]. Also, practices were dichotomized in two equal groups using the volume of practice nurses corrected for practice size, as the workload can affect the study results as well. This resulted in four strata. For each stratum, a separate two-block randomization list was produced to randomize the practices to the control or intervention arm [33]. Two practices were coupled in the randomization procedure to prevent contamination, because one practice nurse worked in both practices. The final allocation of practices is shown in Table 1.

**Table 1:** Randomization strata and the number of practices per stratum of the SeMaS study

	**Low participation in behavioral change training**		**High participation in behavioral change training**	
	**Intervention**	**Control**	**Intervention**	**Control**
Low volume of practice nurses	2	1	2	2
High volume of practice nurses	1	3	2	2

Volume of practice nurses: the number of full time equivalents divided by the number of patients in the practice. Participation in training: percentage of the practice nurses who attended the training for behavioral change to enhance self-management that was provided in 2011 [29]. SeMaS, self-management screening questionnaire.

A representative of the care group DOH consented with participation in the study for all practices. After performing the randomization procedure, we informed the practices about participation in the study.

### Patients

The population of chronically ill patients in the practices consists of approximately 9,100 patients with cardiovascular risk, 3,900 diabetes mellitus patients, 1,500 asthma patients and 900 COPD patients. These patients are regularly seen by the practice nurse. Patients can be included if they are over 18 years of age, have at least one of these chronic conditions and have a checkup appointment with the practice nurse in the inclusion period. Practices will send invitations with information about the study and informed consent forms to patients to participate in the study approximately 4 weeks before the planned checkup appointment of the patient. Patients will be given the option to fill in the questionnaires either digitally through the Internet or on paper. Participating patients will be asked to fill in an evaluative questionnaire one week after their consultation, and the final questionnaire after 6 months.

**Figure 2:**
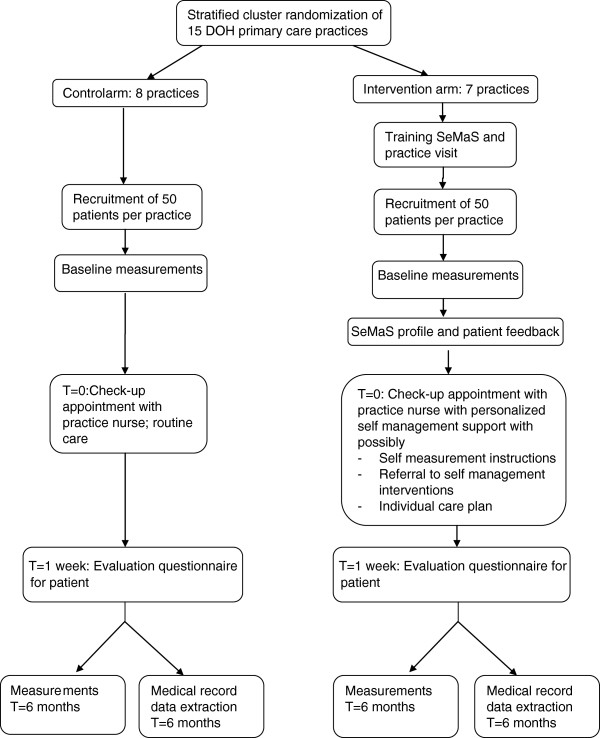
**Flow chart of the self-management screening questionnaire (SeMaS) cluster randomized trial.** Baseline measurements and measurements at time (T) = 6 months include: SeMaS, Patient Activation Measure (PAM-13), perceived competence scale, short test of functional health literacy in adults (S-TOFHLA), rapid assessment of physical activity (RAPA), rapid eating assessment for participants-short (REAP-S), and smoking. DOH, *De Ondernemende Huisarts* (The Innovative General Practitioner).

Medical-record data extraction will be performed by the data management agency of the care group. They can provide anonymous data on the participating patients from the research period. The research team will perform medical-record data analysis. The flow chart of the trial is shown in Figure 2.

### Ethical approval

The study has been reviewed by the local Medical Research Ethics Committee, the CMO region Arnhem-Nijmegen (registration number: 2012/561). The study will be carried out in accordance with the applicable rules concerning the review of research ethics committees and informed consent.

### Self-management screening tool: SeMaS

The SeMaS questionnaire was developed in four steps. First, we performed a broad, systematic literature search to identify reported aspects that are associated with successful or unsuccessful self-management. Second, we held focus groups with professionals from primary care (GPs, psychologist, dietitian, physiotherapist, pharmacist) and patients to identify the most important aspects that would determine the chance of successful self-management, based on their experience and the literature search. The final selection of aspects was made in the stakeholders group, combining the findings of the literature study and focus groups. The aspects were selected if they were mentioned in the focus group, found in the literature, and an instrument was available. Third, we developed a prototype of the SeMaS and tested it in 24 consultations for applicability and readability, resulting in minor adjustments. Fourth, we validated the SeMaS in a test with 200 patients. Patients completed the SeMaS before their control visit with a practice nurse. After two months, patients completed a second questionnaire with SeMaS, reference questionnaires, and an additional questionnaire for process evaluation. Also, medical record data was extracted for process evaluation. Specifically, data were collected on referrals to self-management interventions, and advice or instructions provided to enhance self-management. Analysis of the data resulted in minor adjustments of the SeMaS.

SeMaS is designed to be generally applicable to patients with chronic conditions. It consists of 27 items divided over the relevant aspects of self-management, namely, burden of disease, locus of control, self-efficacy, social support, coping style, anxiety, depression and skills (computer, groups and self-care). A publication of the validation study of SeMaS is in progress.

Screening with SeMaS results in a personal profile on these aspects that are important for self-management, divided into three categories per aspect: 1) capable of (more) self-management, 2) capable of self-management with minor barriers, and 3) major barrier(s) for (more) self-management at this time. These categories were based on the scoring categories of the original instruments, and face validity. SeMaS will support the creation of individual care plans, make it possible to influence barriers for self-management, and support the referral to and participation in self-management activities. The profile will provide an overview of the aspects needing special attention when undertaking self-management activities.

### Intervention

The intervention will be delivered at cluster level. The intervention consists of personalizing self-management support using the results of the SeMaS questionnaire. The results are represented in a report with a graphic profile of the patient and tailored advice to enhance self-management, as described in a manual.

### Training and support of practice nurses in the intervention arm

Intervention practice nurses and GPs will receive a two-hour training session before starting the trial, consisting of a brief introduction to the SeMaS, demonstration of a consultation with a SeMaS report and skills practice using role play. The practice nurses will be specifically instructed on the options for personalized self-management support, such as the creation of individual care plans, options to influence the barriers for self-management, and the referral to self-management interventions. During the training, the practice nurses will receive a manual that indicates which profiles are suitable for self-management, which are suitable with minor barriers, and which are unsuitable for self-management at this time. The manual also contains the instructions for personalized self-management support.

Subsequently, intervention practices will be visited to provide further support in working with the SeMaS. The user manual and examples of reports with suggestions for personalized self-management support will be discussed.

### Self-management support

During the study period, practice nurses of the intervention arm will receive a report with the profile of the patients who filled in the questionnaire. Patients will also receive tailored feedback. Practice nurses are instructed to adjust the delivery of care to the profile. For each factor the manual provides advice to help support the patient in case of a barrier. For example, when a patient experiences low social support, the practice nurse and the patient search for additional social support, or find ways to cope with this low support. For a patient with low self-efficacy, the advice is to set a goal with the patient with a high chance of success, to foster the self-efficacy. When patients show major barriers for self-management, the manual gives instruction and support for the practice nurse to work on this barrier before starting with self-management activities.

When no barrier is present, the practice nurse is advised to create an individual care plan with the patient, and refer to the self-management interventions, if applicable. Also, the manual contains a card with the possible self-management interventions, categorized by the skills that are asked about in the SeMaS questionnaire (computer, group, self-care). The manual was developed by the research team and reviewed by the stakeholders group. The advice was based on the method of actual practice and maintenance, which is used in the care group.

Control practices will deliver care as usual, and will receive the training one year later. Care as usual may include the use of an individual care plan or referral to the self-management interventions, as this is available for the entire care group. Patients will be invited in the same inclusion period as in the intervention arm. They will be asked to fill in the questionnaire, without feedback to themselves or their practice nurse.

### Outcomes

The primary outcome is the difference in patient activation score between baseline and six months between the intervention and control arm, using the PAM-13 [23]. The PAM-13 is an interval-level, uni-dimensional, Guttman-like measure. In the study of Greene and Hibbard the average PAM score was 66.4 (SD 15.4; n = 25,047) [34].

We expect that using the SeMaS instrument will lead to more effective self-management support. Also, we expect that patients will adhere better to the self-management interventions, as they are referred based on the SeMaS profile. To measure whether the self-management support is more successful in the intervention arm than in the control arm, we will perform medical-record data analysis. The outcome measures include the number of completed individual care plans, the number of patients performing self-monitoring, the number of referrals to self-management interventions (group courses and internet coaches), and adherence to these interventions, the number of referrals to informative websites, and the number of consultations in the general practice and emergency care in the study period. We will assess how these data relate to the results on the SeMaS questionnaire at baseline.

Furthermore, we expect that patients who receive personalized self-management support, will be more able to improve their lifestyle than patients receiving care as usual. Lifestyle factors will be measured with the rapid assessment of physical activity (RAPA) and the rapid eating assessment for participants-short (REAP-S) [35,36]. Smoking behavior will be measured with the 10-item Behavior Change Consortium questionnaire [37]. The difference in the score per lifestyle behavior between baseline and time (T)1 will be calculated to assess the change in lifestyle behavior. The secondary outcome measures are defined as the difference in the average score between control and intervention at T0 and T1 for the RAPA and REAP-S questionnaires. For smoking, the outcome is defined as the difference between the numbers of patients smoking in the control and intervention arms at T0 and T1.

#### Outcomes from the evaluation questionnaire one week after consultation

To evaluate whether the SeMaS was discussed, and which actions were undertaken with the patient, an evaluation questionnaire was newly developed. Patients will be asked whether the SeMaS profile was discussed and which of the psychosocial aspects from SeMaS were covered during the consultation. Current self-management activities, and details of whether the patient received information, advice or referral to interventions for lifestyle or self-care, will also be covered in the questionnaire. The participating patients will receive the questionnaire one week after the consultation. These data will be compared in the intervention and control arm.

By triangulation of the data from the medical records, the evaluation questionnaire and the SeMaS profile, we will assess the number and type of SeMaS dimensions discussed with the patients related to the SeMaS scores on these various dimensions. More explicitly, per dimension we will assess whether this was a barrier in the profile, and whether it is mentioned in the medical record and the evaluative questionnaire. If so, this will be scored as proof of attention. We will assess the percentage of patients with a barrier and proof of attention for that dimension/barrier.

#### Qualitative outcomes

The use of SeMaS will be evaluated with the professionals in the intervention arm by semi-structured interviews. Informed consent for these interviews will be obtained. Questions for the interview will be formulated using the barriers and facilitators on different levels of healthcare [38]. Special attention will be paid to the usefulness of the instrument in actual practice and the additional value for improvement of self-management of patients and the creation of individual care plans. The purposive sample size will be determined based on theoretical saturation. Therefore, data review and analysis will be done in conjunction with data collection.

### Sample size

Sample size calculations have been made using the results from three studies on the primary outcome measure, PAM-13 [17,34,39]. In these studies, improvements in PAM scores after intervention vary from 4% to 8% (SD 12 to 17). Based on these studies, we expect to find a difference in PAM score of 6% (SD 14.0) in this study. For the power calculations, we assume an intraclass correlation coefficient (ICC) of 0.05 based on the article of Campbell *et al*. [40]. This article states that an ICC of 0.0 to 0.5 is normal for outcomes in primary care research. To reach a power of 80% and alpha of 0.05, and considering ICC of 0.05, at least 25 patients per practice are needed when using 15 practices. With an expected dropout rate of 33%, 33 patients would be needed per practice, thus, 495 in total. Our secondary outcomes comprise lifestyle measures that will not be applicable to every patient, such as smoking. Therefore, we strive to include 50 patients per practice. Thus, 750 patients will be included in total.

### Analysis

We made an *a priori* analysis plan for the primary analysis of the data, including details about the primary and secondary outcomes, covariates, treatment of missing values, and planned analyses. The outline of the analysis plan will be described here. The primary analysis will be an intention-to-treat analysis to test the hypothesis that patients who received personalized self-management support will be more activated than patients who did not receive personalized self-management support, expressed by the PAM-13 score. Secondary analyses will test the hypothesis that patients who received personalized self-management support will be more able to change their lifestyle in a positive way than patients who did not receive personalized self-management support, as measured with the RAPA, REAP-S and 10-item Behavior Change Consortium questionnaire.

Using analysis of covariance (ANCOVA) (that is, multilevel linear regression with the follow-up score as the outcome and the baseline score as a covariate), the difference in scores on PAM-13 in the subgroups at T = 0 and T = 6 months will be examined in the control and intervention arm. Subsequently, we will analyze the impact of the intervention on predefined subgroups as measured by the SeMaS. We defined three subgroups: patients who are ready for self-management, patients who can undertake self-management with minor barriers, and patients with severe barriers to self-management, by calculating an overall score on the SeMaS questionnaire.

Each secondary outcome will be analyzed with a multilevel multivariate covariance regression model, with the analyst blind to practice allocation to trial arms. Covariates that will be controlled for are: age, gender, chronic condition (from the medical-record data), social support (as measured with the SeMaS), diagnosis of depression based on the International Classification of Primary Care (ICPC) code and health literacy (as measured with the Short Test of Functional Health Literacy in Adults (S-TOFHLA)) [41,42], and the baseline scores. All statistical analyses will be performed using SPSS software (version 20, IBM Corp.), SAS (version 9.2, SAS Institute Inc.) or MLWIN (version 2.28, University of Bristol).

We will also perform a per protocol analysis of data from patients who received a referral to a self-management intervention. We will examine adherence to the self-management intervention program, comparing patients from intervention practices with control. Also, we will test the effect of the intervention on process outcomes (filled in individual care plans, the number of referrals to self-management interventions, and the number of consultations in the general practice and emergency care) using multilevel multivariate regression techniques.

The effect of cluster will be determined by performing a multilevel regression analysis without explaining variables. With this analysis, we can determine the variance at the different levels. Subsequently, we can determine the intraclass correlation of this study:

ICC=Clustervariance/Clustervariance+Patients'variance

We will qualitatively analyze the interviews with healthcare professionals. The interviews will be transcribed verbatim. The transcripts will be analyzed by open coding at macro level using predefined main codes according to the barriers and facilitators at different levels of healthcare, as proposed by Grol and Wensing [38]. These levels are: innovation, individual professional, patient, social context, organizational structure, and economical and political context. After the open coding, the research team will group themes and subthemes for each level of healthcare. The program Atlas.ti (version 6, ATLAS.ti Scientific Software Development GmbH) will be used for analysis.

## Discussion

This manuscript presents the protocol for a cluster randomized clinical trial of personalized self-management support, using the SeMaS questionnaire in patients with chronic conditions, in primary care. By carrying out this study, scientific evidence is built for the effectiveness of personalized self-management support. As stated by Hibbard *et al*., patient activation is a significant predictor of healthcare costs [43]. Therefore, if effects of the personalized self-management support on the PAM-13 scores are found, this will also indicate effects on costs.

### Limitations of this study

This study is carried out in the DOH care group. This care group consists of group practices in primary care, and has an innovative mindset. Therefore, the implementation of SeMaS in other general practices will need guidance from experts. Also, implementation in other practices will need to be evaluated. Recruitment bias may be caused by the response of patients with certain characteristics, including current self-management status, and health literacy. Due to privacy legislation, it will not be possible to analyze the characteristics of non-responders.

### Trial status

At the time of submission of this study protocol, we are recruiting patients for the trial.

## Abbreviations

ANCOVA: Analysis of covariance; COPD: Chronic obstructive pulmonary disease; DIEP: Diabetes interactive education program; DOH: De Ondernemende Huisarts; GP: General practitioner; heiQ: Health education impact questionnaire; ICC: Intraclass correlation coefficient; ICPC: International classification of primary care; PAM: Patient activation measure; RAPA: Rapid assessment of physical activity; REAP-S: Rapid eating assessment for participants-short; SeMaS: Self-management screening questionnaire; S-TOFHLA: Short test of functional health literacy in adults.

## Competing interests

The authors declare that they have no competing interests.

## Authors’ contributions

NE drafted the manuscript. JL, MW, IS and AJ critically revised the manuscript. All authors have been involved in the design of the study. All authors have read and approved the final manuscript.
